# Inhibitory Effects of Ginsenoside Rb1 on Early Atherosclerosis in ApoE-/- Mice via Inhibition of Apoptosis and Enhancing Autophagy

**DOI:** 10.3390/molecules23112912

**Published:** 2018-11-08

**Authors:** Ping Zhou, Weijie Xie, Yun Luo, Shan Lu, Ziru Dai, Ruiying Wang, Xuelian Zhang, Guang Li, Guibo Sun, Xiaobo Sun

**Affiliations:** 1 Institute of Medicinal Plant Development, Peking Union Medical College and Chinese Academy of Medical Sciences, Beijing 100193, China; zhoup0520@163.com (P.Z.); xwjginseng@126.com (W.X.); ly20040423@126.com (Y.L.); lushanluna@163.com (S.L.); athenadai219@163.com (Z.D.); wangruiying8866@163.com (R.W.); xlZhang2022@163.com (X.Z.); lhbg311@hotmail.com (G.L.); 2 Beijing Key Laboratory of Innovative Drug Discovery of Traditional Chinese Medicine (Natural Medicine) and Translational Medicine, Beijing 100193, China; 3 Key Laboratory of Bioactive Substances and Resource Utilization of Chinese Herbal Medicine, Ministry of Education, Beijing 100193, China; 4 Key Laboratory of Efficacy Evaluation of Chinese Medicine against Glycolipid Metabolism Disorder Disease, State Administration of Traditional Chinese Medicine, Beijing 100193, China

**Keywords:** ginsenoside Rb1, atherosclerosis, inflammation, apoptosis, autophagy

## Abstract

Inflammation is a major contributing factor to the progression of atherosclerosis. Ginsenoside Rb1 (Rb1), an active saponin of *Panax notoginseng*, has been found to exert beneficial effects on inflammation and oxidative stress. This study investigated the ability of Rb1 to inhibit the formation of atherosclerotic plaques and the potential mechanisms. In this study, the effects of Rb1 on the development of atherosclerosis were investigated in ApoE-/- deficient mice fed with a western diet. Mice were intragastrically administrated with Rb1 (10 mg/kg) for 8 weeks. This study is that ginsenoside Rb1 exerted an inhibitory effect on early atherosclerosis in ApoE-/- mice via decreasing body weight and food intake daily, upregulating the lipid levels of serum plasma, including those of TC, TG and LDL-C and HDL-C and reducing the atherosclerotic plaque area, suppressing inflammatory cytokines (levels of IL-1β, IL-6 and TNF-α) in the serum of ApoE-/- mice, changing the expression levels of BCL-2, BAX, cleaved caspase-3 and cleaved caspase-9 and weakening apoptosis associated with anti-inflammatory activity. Hence, all these effects against atherosclerosis were tightly associated with regulation of necrosis or apoptosis associated with anti-inflammatory activity. Additionally, the results found that ginsenoside Rb1 increased autophagy flux to inhibit apoptosis via acceleration of autophagy by promoting transformation of LC3 from type I to type II in high-fat diet-induced atherosclerosis in ApoE-/- mice. This finding, along with those of the previous study, provides evidence that Rb1 promotes the process of autophagy to protect against atherosclerosis via regulating BCL-2 family-related apoptosis. These results indicate that Rb1 exhibits therapeutic effects in atherosclerosis by reversing the imbalance between apoptosis and autophagy.

## 1. Introduction

Atherosclerosis, a disease of the large arteries, is the principal cause of heart disease and stroke [1]. Accumulating evidence has demonstrated that inflammation plays a critical role in coronary artery disease (CAD) and other atheromatous diseases [2]. The production of several inflammatory cytokines, including tumour necrosis factor (TNF)-a, interleukin (IL)-1β, and IL-6, and several endothelial adhesion molecules, such as vascular cell adhesion molecule-1 (VCAM-1) and intracellular adhesion molecule-1 (ICAM-1), is involved in the pathogenesis of atherosclerosis [3,4]. Stimulation by high levels of inflammatory factors results in monocyte adhesion and differentiation into macrophages; the macrophages then become lipid-laden and transform into foam cells [5]. A critical sign of atherosclerosis is the accumulation of foam cells in lesions, and, in particular, the recruitment of macrophages into plaques is crucial for the process of atherosclerosis. Numerous studies have indicated that many types of apoptosis resulting from inflammation are pathological changes of atherosclerosis, including apoptosis of endothelial cells, vascular smooth muscle cells and even foam cells, and that inhibiting apoptosis can alleviate the development of atherosclerosis [6,7,8].

Recently, autophagy, a conserved response mechanism by which cells obliterate macromolecular substances and damaged organelles, has been reported to be play an important role in multiple cellular processes, including starvation, oxidative stress, inflammatory reactions, immune responses and a wide range of diseases [9,10]. Moreover, accumulating studies show that autophagy induction is a therapeutic target in atherosclerosis [11,12]. Numerous studies have suggested that the autophagy-lysosome system is damaged in atherosclerosis models [13]. Under normal circumstances, a balance exists between apoptosis and autophagy that maintains cellular environmental homeostasis; this balance is destroyed in vascular diseases such as atherosclerosis. Thus, therapeutic strategies that regulate apoptosis and autophagy may be effective for atherosclerosis [14].

Vascular endothelial cells require mitochondrial homeostasis to maintain their functionality. Not only is mitochondrial metabolism required to produce ATP for cellular function, but mitochondria are also critically important for the close interactions among oxidative stress, inflammation, apoptosis, and autophagy. Ginsenoside Rb1 has been proven to exert protective effects against mitochondrial pathway apoptosis by regulating BCL-2 family proteins [15,16].

The BCL2 proteins antagonize apoptosis via a combination of BAX (BCL2-associated X protein) and heterodimer formation. The phosphorylation of BCL2 at Ser70 enhances the effect of apoptosis inhibition through interaction with BAX and BAD (BCL2-associated death promoter). In contrast, BCL2 also modulates autophagy by regulating BECN1/beclin 1. Furthermore, autophagy is activated by the dissociation of BCL2 from BECN1 due to BCL2 phosphorylation [17]. These studies indicate that BCL2 balances apoptosis and autophagy to maintain cellular environmental homeostasis.

**Figure 1:**
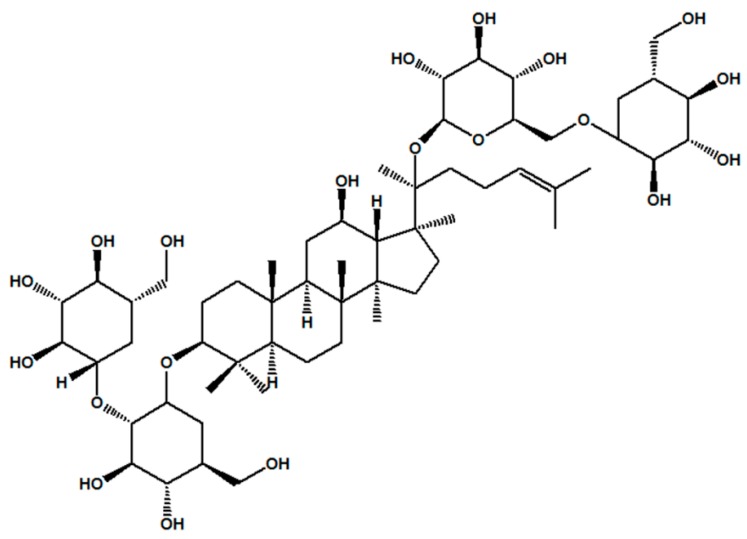
Molecular structure of ginsenoside Rb1.

Ginseng has been used worldwide as a popular traditional medicine for years, most notably in East Asia, including China, Japan and Korea [18]. Among more than 30 kinds of ginsenoside isolated from *Panax ginseng* C. A. Mey, Rb1 (chemical structure shown in Figure 1) is the essential active component contributing to many of the pharmacological effects of *P. ginseng*. A recent pharmacological study suggested that Rb1 exerts a protective effect against apoptosis in myocardial cells [19] and in ischaemia-reperfusion [20]. Rb1 has also been reported to increase insulin sensitivity to clinically alleviate diabetes and its complications [21,22]. Recent studies have demonstrated that Rb1 has oestrogenic properties, as observed in human breast cancer MCF-7 cellsb [23]. Ginsenosides Rb1 have also been reported to exert hepatoprotective effects in tert-butyl hydroperoxide-induced liver injury [24]. Ginsenoside Rb1 has been proven to protect against 6-hydroxydopamine-triggered oxidative stress by upregulating haeme oxygenase-1 expression [25], the beneficial effects of which are associated with free-radical scavenging.

Our previous study found that ginsenoside Rb1 dramatically attenuates TNF-α-induced HUVEC injury. The protective effect was associated with the suppression of inflammation and oxidative stress and the alleviation of mitochondrial injury. Moreover, this anti-apoptotic effect was exerted by regulation of the BCL-2 family proteins. The results indicate that once these effects of ginsenoside Rb1 are validated in animal studies and clinical trials, Rb1 might be an attractive drug candidate for the prevention and treatment of atherosclerosis [26].

Thus, the present study was designed to further investigate the protective role of Rb1 against atherosclerosis using the well-defined classical ApoE-/- mouse atherosclerotic model. We also examined whether Rb1 inhibits the formation of atherosclerotic plaques and restores the balance between apoptosis and autophagy.

## 2. Materials and Methods

### 2.1. Reagents and Materials

Ginsenoside Rb1 (molecular weight = 1109, purity > 98%) was purchased from Shanghai Winherb Medical S&T Development (Shanghai, China). Oil Red O and commercial kits for detecting total cholesterol (TC), triglycerides (TG), cholesterol (CHOL), low-density lipoprotein cholesterol (LDL-C) and high-density lipoprotein cholesterol (HDL-C) were obtained from Biosino Biotechnology & Science Inc. (Beijing, China). Enzyme-linked immunosorbent assay (ELISA) kits for measuring mouse IL-6 and TNF-α were obtained from Beijing Expandbiotech Co., Ltd. (Beijing, China). Mouse MCP-1 and IL-1β ELISA kits and a kit for terminal deoxynucleotidyl transferase biotin-dUTP nick end labelling (TUNEL) were purchased from Roche Diagnostics GmbH (Mannheim, Germany). DAPI dihydrochloride staining reagent was provided by Beyotime Institute of Biotechnology (Beijing, China). A BCA protein assay kit, protease inhibitor cocktail and enhanced chemiluminescence kit were obtained from CWbiotech (Beijing, China). Mammalian protein extraction kits were purchased from Santa Cruz Biotechnology, Inc. (Santa Cruz, CA, USA). Primary antibodies were purchased from Abcam (Cambridge, UK), and secondary antibodies were obtained from Santa Cruz Biotechnology, Inc. Dimethyl sulfoxide (DMSO) was from Sigma Aldrich (St. Louis, MO, USA).

### 2.2. Animals

Male ApoE-/- mice with a C57BL/6 N background and C57BL/6 N mice (weighing 18 g to 20 g, 6 weeks old) were obtained from the Experimental Animal Centre of Peking University of Medical Sciences (Beijing, China). All interventions and animal care methods were conducted according to the Guidelines and Policies for Animal Surgery offered by the Laboratory Animal Ethics Committee of the Institute of Medicinal Plant Development (Peking Union Medical College, Beijing, China). The mice were kept in a temperature-controlled facility (temperature: 24 °C to 25 °C, humidity: 55%) with a 12 h light/12 h dark photoperiod, and food and water were freely available.

### 2.3. Experimental Protocol

After one week of feeding acclimation, twenty male ApoE-/- mice were randomly divided into two groups, a model group and an Rb1 administration group (10 mice for each group). Twenty male C57BN/L6 mice were used for a control group and an Rb1 administration group. ApoE-/- mice were fed a “western diet” for 12 weeks, consisting of 78.85% of basic mice maintain feed, 21% of fat and 0.15% of cholesterol. The Rb1 group was administered Rb1 in saline intraperitoneally at a dose of 10 mg/kg/day from the 4th week to the 12th week, whereas the C57BN/L6 control mice and ApoE-/- model mice received the same volume of vehicle. Drug administration was conducted once daily at approximately 9 a.m.

The body weights of the mice were measured at the 0, 2, 4, 6, 8, 10 and 12 weeks. The mice were fasted 12 h at the beginning and end of the experiment; the eyes were extracted and blood was drawn at week 12. Cervical dislocation was used to euthanize the mice, and the hearts with aortic arch were removed and fixed in 4% formalin. The upper portions of the hearts with the aortic cusps were embedded in paraffin, and 6 μm-thick sections were cut for immunofluorescence staining, Oil Red O staining or TUNEL staining.

### 2.4. Serum Analysis of Lipids and Plasma Cytokines

The levels of serum lipids, including TC, TG, LDL-C, and HDL-C, were detected using the corresponding kits with a Hitachi 7600 Automatic Biochemistry Analyzer (Tokyo, Japan) following the manufacturer’s instructions. Serum levels of cytokines, including IL-1β, IL-6, MCP-1 and TNF-α, were determined with enzyme-linked immunosorbent assay (ELISA) kits according to the manufacturer’s instructions.

### 2.5. Oil Red O Staining

After twelve weeks of Rb1 (10 mg/kg via i.p.) administration, the upper portions of hearts embedding in optimum cutting temperature (OCT) compound, the aortic roots of each mouse were sliced into 60 cross-sections (6 μm) in the aortic valve. The resultant aortic sinus cryosections were investigated by means of oil red O staining using Image-Pro Plus (Media Cybernetics, Silver Springs, MD, USA) software. The area of atherosclerotic plaque in each aortic section was evaluated based on oil red-O-staining using Image-Pro Plus software. At least five samples of each animal from one treatment group were used in the subsequent analyses, and the lesions were estimated from 10 different mice.

### 2.6. Immunohistochemical Analysis

Immunohistochemistry experiments were performed to detect cleaved caspase-3 expression in the aortic arch. In brief, the prepared tissue sections obtained from C57 or ApoE-/- mice (with Rb1 administration or not) were incubated with a caspase-3 (ab13847) primary antibody (5 µg/mL) at 4 °C overnight. After washing, the arterial sections were incubated with anti-rabbit IgG goat antibodies (1:100) for 40 min at room temperature with anti-rabbit IgG goat antibodies (1:200) followed by incubating with 0.5 g/L diaminobenzidine for 10 min. ImageJ software was used to quantify caspase-3 expression.

### 2.7. Detection of DNA Fragmentation

A TUNEL staining kit (Roche 11 684 817 910) was used to determine the DNA fragmentation in the arteries in accordance with the manufacturer’s instructions. Briefly, the embedded aorta sections were maintained at room temperature for 10 min followed by rinsing twice using PBS to remove the OCT compound. The TUNEL working solution was mixed according to the protocol, and then 50 μL of the TUNEL mixture was used to cover the samples at 37 °C in a wet box for 60 min. The liquid around the tissue was dried, followed by the addition of 50 µL transformation-POD (vial 3) to the tissue, which was maintained in a wet box at 37 °C for 60 min. The tissues were rinsed 3 times with PBS; then, 50 µL of DAB substrate was added, and the tissues were incubated at room temperature (10 min). After rinsing thrice for 5 min in PBS, the samples were stained with haematoxylin for 5 min and then observed under a light microscope. The percentage of TUNEL-positive cells was calculated using ImageJ software.

### 2.8. Transmission Electron Microscopy

Samples were fixed in 2.5% glutaraldehyde in 0.1 M sodium cacodylate buffer (pH 7.2) for 2 h at room temperature followed by rinsing with phosphate buffer four times for 60 min. Subsequently, 1% osmic acid was used to fix each sample at 4 °C for 2 h. After staining with a 2% aqueous solution of uranyl acetate for half an hour, a succession of graded ethanol concentrations was used to dehydrate the samples before embedding them in PolyBed. Blocks were sectioned at a thickness of 80 nm, stained with uranyl acetate and lead citrate and then viewed under a transmission electron microscope (Tecnai G2, FEI, Hillsboro, OR, USA).

### 2.9. Western Blot Analysis

Aorta tissues from different groups were homogenized with saline at a ratio of 1:9 (mg:μL). The samples were centrifuged at 7000 rpm for 5 min. Then, tissue-protein extraction reagent containing 1% proteinase inhibitor and phosphatase inhibitor cocktail was added to extract the proteins, followed by incubation on ice for 30 min. The protein concentration was detected with a BCA kit (CWbiotech, Beijing, China). Cytoplasmic and nuclear protein samples were separated by protein extraction kits containing 1% phenylmethylsulfonyl fluoride (CoWin Bioscience Co., Ltd., Beijing, China). Western blot analysis was performed according to previously described methods [26]. Briefly, equal amounts of proteins were subjected to 8–12% Bis-Tris gel electrophoresis in a Bio-Rad slab gel apparatus (Mini-PROTEAN Tetra cell; Bio-Rad, Hercules, CA, USA) and electrophoretically transferred to nitrocellulose membranes. The membranes were incubated overnight at 4 °C with the following primary antibodies: β-actin (sc-8432), lamin B (sc-6216) against LC3B (#2775), BECN1 (ab207612), and sequestosome 1 (SQSTM1)/p62 (ab56416). The membranes were subsequently incubated with horseradish peroxidase-conjugated secondary antibodies at room temperature for 2 h. After washing with Tris-buffered saline and Tween 20 (TBST) three times for 45 min, the protein blots were incubated with an enhanced chemiluminescence (ECL) reagent. Protein expression levels were observed using Image Lab Software (Bio-Rad). At least three separate experiments were performed with different lysates to confirm the changes in protein levels, and densitometry analysis was performed.

### 2.10. Statistical Analysis

The data are expressed as the mean ± the standard error (SE) of the mean in each group. Differences between groups were tested by one-way ANOVA followed by the Student-Newman-Keuls test with GraphPad Prism 5.0 software (Inc., La Jolla, San Diego, CA, USA). Statistical significance was considered to be “*p* < 0.05”.

## 3. Results

### 3.1. Ginsenoside Rb1 Decreased Body Weight and Food Intake in ApoE-/- Mice Fed a High-Fat Diet

**Figure 2:**
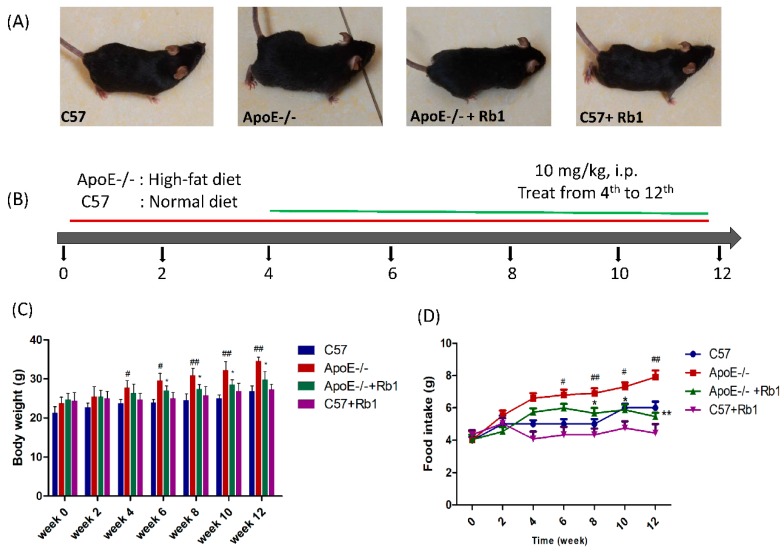
Rb1 suppressed body weight and food intake in ApoE-/- mice fed a high-fat diet. (**A**) Status of C57 and ApoE-/- mice in each group after Rb1 or vehicle administration. (**B**) C57 mice fed a normal diet and ApoE-/- mice fed a high-fat diet. ApoE-/- mice and C57 mice were treated intraperitoneally with Rb1 (10 mg/kg i.p.) or the same volume of vehicle in saline from the 4th week to the 12th week. (**C**) Body weight of the mice in all groups during 12 weeks of a high-fat diet. (**D**) Food intake of mice in all groups during 12 weeks of treatment with a high-fat diet. The data are expressed as the mean ± the standard error (SE) of the mean in each group (*n* = 10). *^##^ p* < 0.01, *^#^ p* < 0.05 vs. C57 control; * p < 0.05, ** p < 0.01 vs. ApoE-/- model.

As shown in Figure 2C,D, body weight and food intake gradually and dramatically increased in the HFD-fed ApoE-/- mice, especially after the 4th week, compared with those of the normal diet-fed C57 mice. However, body weight and food intake were reduced significantly by Rb1 (10 mg/kg) administered intraperitoneally in HFD-fed ApoE-/- mice beginning on week 6 and continuing until the end of the experiment (Figure 2C,D). No side effects, such as dysentery, were observed.

### 3.2. Ginsenoside Rb1 Reduced Atherosclerotic Development in ApoE-/- Mice

**Figure 3:**
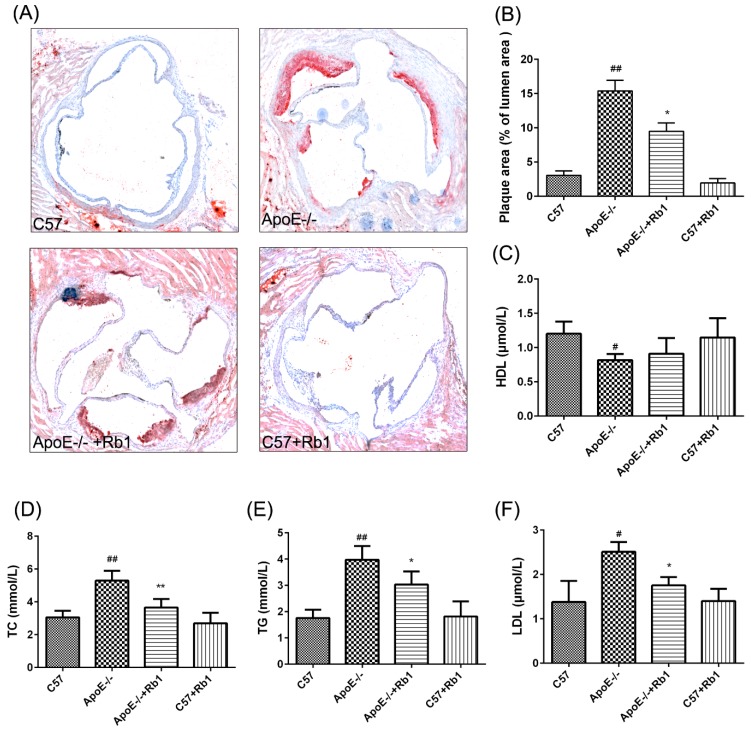
Rb1 reduced atherosclerotic development in ApoE-/- mice. (**A**) Atherosclerotic plaque formation assessed using Oil Red O staining. (**B**) Quantification of plaque areas using ImageJ software. (**C**) HDL-C levels, (**D**) TC levels, (**E**) TG levels, and (**F**) LDL-C levels. Each bar represents the mean ± the standard error (SE) of the mean in each group (*n* = 10). *^##^ p*< 0.01, *^#^ p* < 0.05 vs. C57 control; * p < 0.05, ** p < 0.01 vs. ApoE-/- model.

Lipid deposition was measured to evaluate the effect of Rb1 administration on the aortic root. The results of Oil Red O staining showed that scarcely any visible lesions were present in the C57 group treated with saline or Rb1; in contrast, the experimental ApoE-deficient mice had prominent plaque areas compared with the control mice. However, treatment of ApoE-/- mice with Rb1 substantially attenuated the atherosclerotic plaque area (Figure 3A,B).

Serum plasma lipid levels, including those of TC, TG and LDL-C and HDL-C, were detected with corresponding biochemical kits at the end of 12 weeks. Circulating levels of TC, TG and LDL-C were notably upregulated in ApoE-/- mice, and Rb1 administration significantly reduced these three indicators (Figure 3D–F). Additionally, there was no difference in HDL-C levels between the ApoE-/- mice treated with and without Rb1 (Figure 3C).

### 3.3. Ginsenoside Rb1 Suppressed Inflammatory Cytokines in the Serum of ApoE-/- Mice

**Figure 4:**
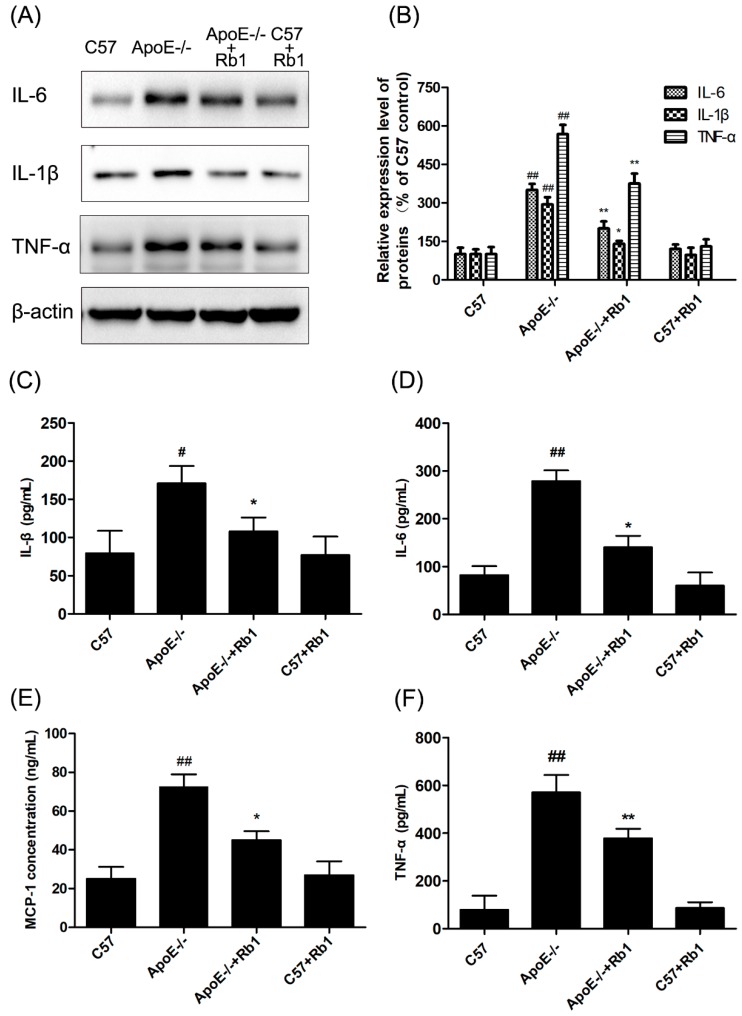
Rb1 suppressed inflammatory cytokines in the serum of ApoE-/- mice. (**A**) Levels of IL-1β, IL-6 and TNF-α as measured by western blot analysis. (**B**) Quantitative analysis was performed on the corresponding bands. (**C**) Effect of Rb1 on IL-1β levels as measured by ELISA. (**D**) Effect of Rb1 on IL-6 levels as measured by ELISA. (**E**) Effect of Rb1 on TNF-α levels as measured by ELISA. (**F**) Effect of Rb1 on TNF-α levels as measured by ELISA. The data are expressed as the mean ± the standard error (SE) of the mean in each group (*n* = 3). *^##^ p* < 0.01, *^#^ p* < 0.05 vs. C57 control; * p < 0.05, ** p < 0.01 vs. ApoE-/- model.

Increased levels of various inflammatory factors in the circulation cause vascular damage, which plays a critical role in the pathogenesis of atherosclerosis. To test the anti-inflammatory actions of Rb1, the levels of IL-1β, IL-6, MCP-1 and TNF-α in the serum were measured using corresponding ELISA kits. In the Rb1 group, the levels of these four inflammatory cytokines were notably decreased compared with those in the ApoE-/- group (Figure 4C–F). Furthermore, western blot analysis was also conducted to determine the levels of these four indicators of protein expression in aortic arch tissue from the different groups. As expected, Rb1 effectively suppressed the production of these four indicators in HFD-fed ApoE-/- mice (Figure 4A,B).

### 3.4. Ginsenoside Rb1 Attenuated the Percentage of Apoptotic Cells in the Aorta of ApoE-/- Mice

**Figure 5:**
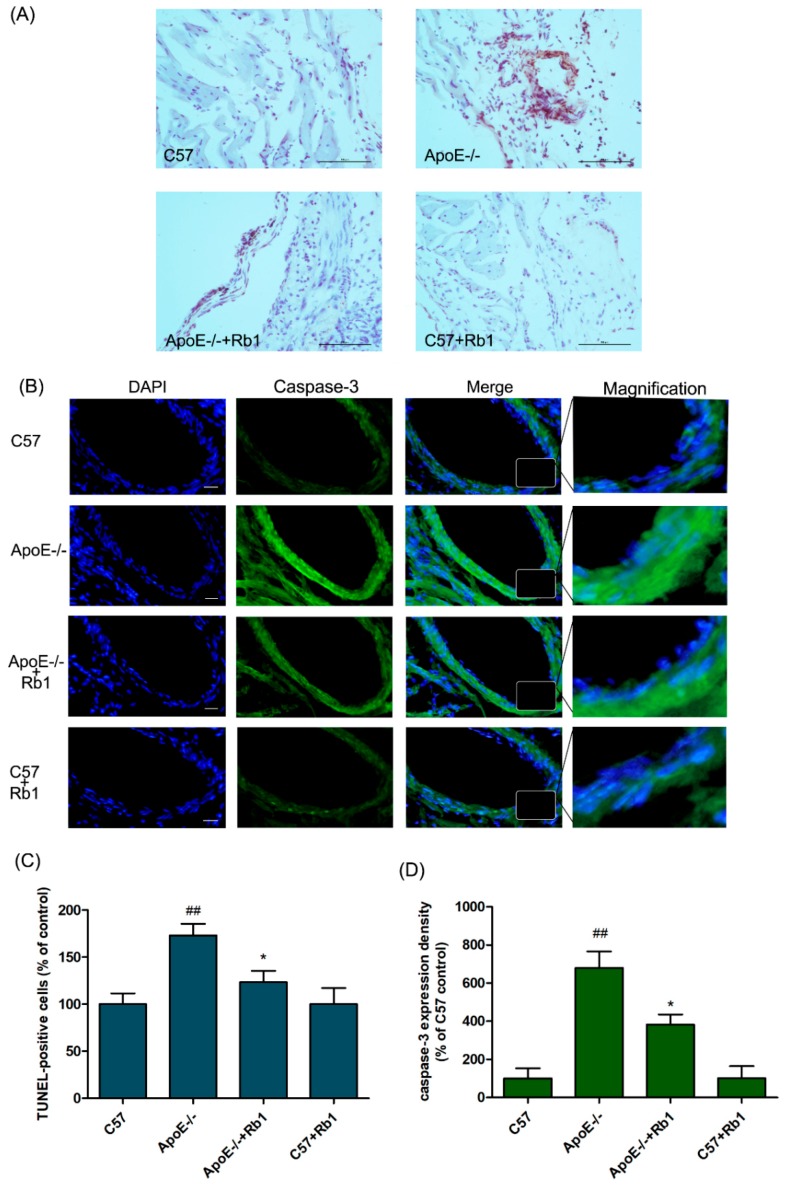
Rb1 attenuated the percentage of apoptotic cells in the aorta of ApoE-/- mice. (**A**) TUNEL staining in the aorta for each group; scale bar, 100 µm. (**B**) Caspase-3 as expression detected by immunohistochemical staining and DAPI; scale bar, 80 µm. (**C**) quantitative analysis of the percentage of TUNEL-positive cells. (**D**) Quantitative analysis of cleaved caspase-3 expression. The data are expressed as the mean ± the standard error (SE) of the mean in each group (*n* = 3). *^##^ p* < 0.01 vs. C57 control; * p < 0.05 vs. ApoE-/- model.

Apoptosis of multiple cell types, such as endothelial cells, is responsible for atherosclerotic vascular damage [27]. In our study, Rb1 administration (10 mg/kg i.p.) dramatically suppressed the percentage of apoptotic cells in the mouse aorta (Figure 5A,C), as determined by TUNEL staining and analysis. In addition, the activation of caspase-3, a representative pro-apoptotic factor, was also detected. As shown in Figure 5B,D, the caspase-3 activation level in the aorta of ApoE-/- mice was markedly upregulated compared with that in the C57 control mice. Rb1 treatment notably inhibited caspase-3 activation. These results suggest that Rb1 treatment in vivo inhibits atherosclerosis development.

### 3.5. Ginsenoside Rb1 Regulated the Expression Levels of Apoptosis-Related Proteins in the Aorta of ApoE-/- Mice

**Figure 6:**
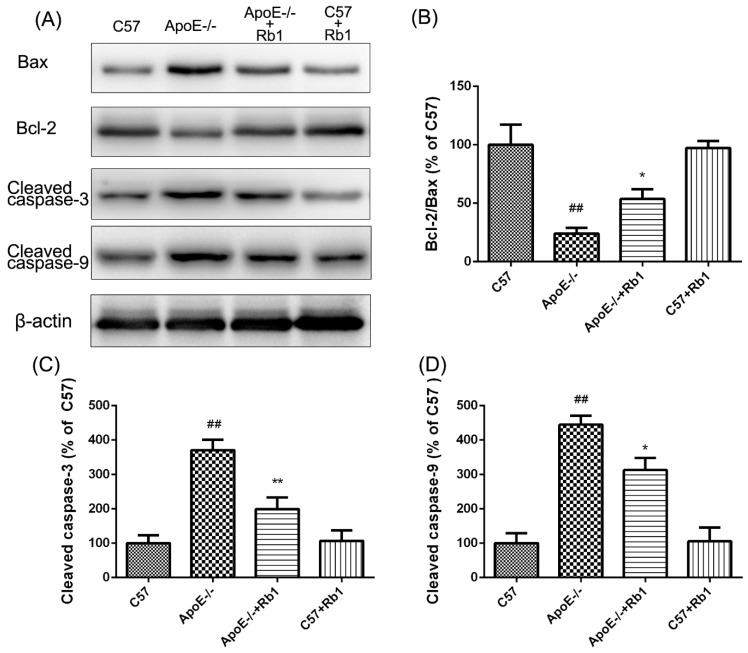
Rb1 regulated apoptosis-related protein expression in the aorta of ApoE-/- mice. (**A**) The levels of Bcl-2/Bax, cleaved caspase-3 and cleaved caspase-9 were determined by western blot analysis. (**B**) Quantitative analysis of the Bcl-2/Bax expression ratio. (**C**) Quantitative analysis of cleaved caspase-3 expression. (**D**) Densitometric analysis was performed to quantify cleaved caspase-9 expression. The data are expressed as the mean ± SD the standard error (SE) of the mean in each group (*n* = 3). *^##^ p* < 0.01 vs. C57 control; * p < 0.05, ** p < 0.01 vs. ApoE-/- model.

To further verify the protective effect of Rb1 against apoptosis during the development of atherosclerosis, western blot analysis was performed. The activation of the caspase cascade is essential in the initiation of apoptosis in diverse biological processes. Consistent with the results of fluorescein-based active caspase-3 staining, western blot analysis displayed an increase in caspase-3 activation in the aorta of ApoE-/- mice; in addition, cleaved-caspase-9 expression levels were enhanced, an effect that could be inhibited by the administration of Rb1 (Figure 6).

Bcl-2 and its binding protein Bax play a pivotal role in the regulation of various apoptotic processes related to vascular injury [28]. As shown in Figure 6, the expression levels of the cytoprotective protein Bcl-2/Bax were decreased in the aorta of HFD-fed ApoE-/- mice; this effect was reversed by Rb1 treatment. Taken together, these results suggest that Rb1 can increase anti-apoptotic protein expression and reduce the expression of pro-apoptotic proteins to protect against cell apoptosis in the aorta.

### 3.6. Ginsenoside Rb1 Administration Accelerated Autophagy in the Aorta of ApoE-/- Mice

Our previous study showed that promoting the autophagy of endothelial cells is conducive to inhibiting apoptosis and thus exerting an anti-atherosclerotic function [29]. Moreover, a recent study suggested that ginsenoside Rb1 exerts an arterial protective effect via enhancing autophagy and lipid metabolism [30]. Thus, in our study, to assess the possible mechanism by which Rb1 suppresses apoptosis in the aorta, autophagosome formation was monitored by transmission electron microscopy, which is the gold-standard method for autophagy detection.

**Figure 7:**
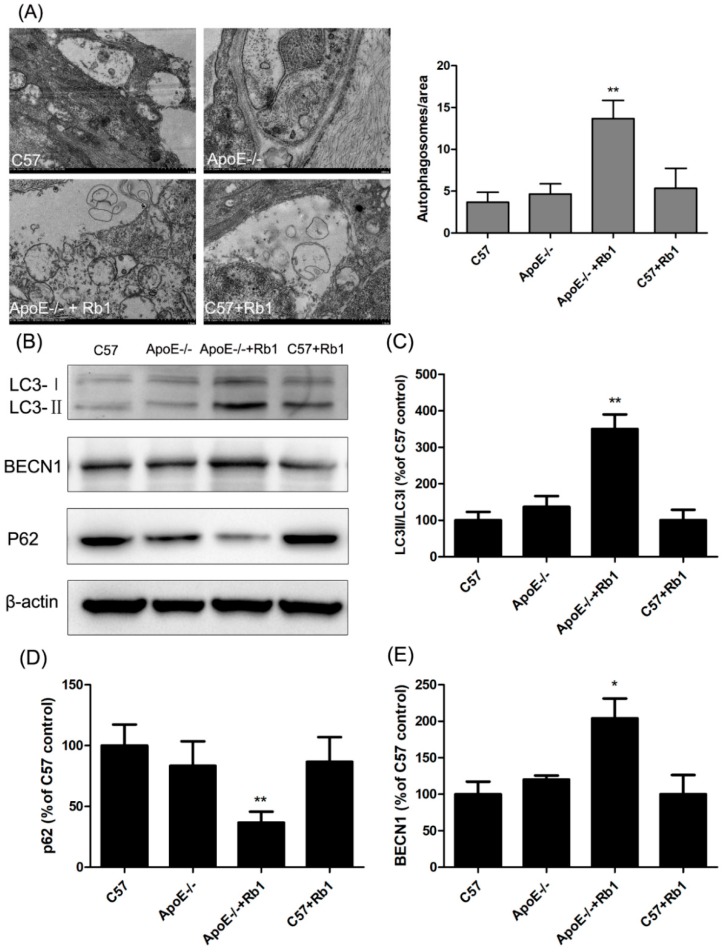
Rb1 administration accelerated autophagy in the aorta of ApoE-/- mice. (**A**) Ultrastructure of endothelial cells of the aorta in each group as revealed by transmission electron microscopy. (**B**) The membrane type of LC3 as well as BECN1 and p62 expression were detected by western blot analysis. (**C**) Quantitative analysis of the LC3-II/LC3-I ratio, (**D**) p62 and (**E**) BECN1. The data are expressed as the mean ±the standard error (SE) of the mean in each group (*n* = 3). * p < 0.05, ** p < 0.01 vs. ApoE-/- model.

Surprisingly, as shown in Figure 7A, plentiful autophagosomes were present in Rb1-treated ApoE-/- mice. To confirm whether administration of Rb1 induced autophagy in the aorta, we examined the membrane type of LC3 as well as BECN1 and p62 expression. As illustrated in Figure 7B–E, Rb1 treatment dramatically promoted the LC3II/LC3I ratio, BECN1 expression and p62 degradation. These results demonstrated that Rb1 administration accelerated autophagy in the aorta of ApoE-/- mice.

## 4. Discussion

Atherosclerosis is a progressive disease characterized by the accumulation of lipids and fibrous elements in the large arteries. In addition, inflammation-triggered endothelial cell injury, which is the initial step in the development of atherosclerotic lesions, is also considered to lead to apoptosis in all cell types both in humans and in animal models [27]. Thus, regulating apoptosis, a critical process occurring in atherosclerosis, to mitigate atherosclerosis is often indispensable.

The species *Panax ginseng*, *Panax quinquefolius* and *Panax notoginseng* in the *Panax* genus have been used as medicinal plants for over 5000 years [31,32]. Their therapeutic effects in multiple diseases, such as neurodegenerative disorders and cardiovascular diseases, have been shown in recent experimental and clinical studies [33]. Ginsenoside Rb1, one of the most abundant and important components isolated from the Panax genus, has been shown to exert numerous beneficial effects on the human body, including effects on the central nervous system, cardiovascular system and immune system, and to have anti-tumour activity [34,35]. These effects are dependent on the anti-inflammatory response, specifically the regulation of the process of autophagy and the inhibition of apoptosis.

Our previous study indicated that ginsenoside Rb1 significantly mitigates TNF-α-induced vascular endothelial cell injury, an effect that involves the suppression of inflammation, the promotion of mitochondrial function and the exertion of anti-apoptotic effects via regulation of BCL-2 family proteins. The results suggest that if these effects of ginsenoside Rb1 are validated in animal studies and clinical trials, Rb1 might be an attractive drug candidate for the prevention and treatment of atherosclerosis. Therefore, in the present study, we investigated the protective effects of Rb1 against high-fat diet-induced atherosclerotic plaque formation in ApoE-/- mice and found that Rb1 could prevent pathological changes in the aortic arteries.

Increased levels of various inflammatory factors in the circulation that play a critical role in the pathogenesis of atherosclerosis, including IL-1β, IL-6, MCP-1 and TNF-α, were detected. However, Rb1 effectively suppressed the production of these four indicators in the serum of HFD-fed ApoE-/- mice, which is consistent with the effects observed on endothelial cells in vitro.

Moreover, in the high-fat diet-fed ApoE-/- mice, DNA fragmentation was observed in the aorta, and caspase-3, which is considered a vital indicator that regulates apoptosis development, was also detected. Interestingly, administration of Rb1 decreased the cleavage of caspase-3 and caspase-9. The Bcl-2 family proteins have been reported to be involved in caspase-dependent apoptosis. Bcl-2 and its binding protein Bax play a pivotal role in the regulation of various apoptotic processes related to vascular injury. In our study, the expression levels of the cytoprotective proteins Bcl-2 and Bax were decreased in the aorta of HFD-fed ApoE-/- mice, an effect that was reversed by Rb1 treatment. In contrast, BCL2 has also been reported to modulate autophagy by regulating BECN1/beclin 1. In recent years, numerous studies have suggested that the regulation of autophagy and apoptosis is often correlated, which has attracted considerable attention [36]. It is widely believed that autophagy plays a vital protective action, inducing atherosclerotic cells to survive in response to various stressful conditions [37,38]. It has been proven that upregulating autophagy inhibits ox-LDL-induced apoptosis in endothelial cells to restore the balance between autophagy and apoptosis [29]. The present study also illustrated that Rb1 accelerated the formation of autophagosomes, upregulated LC3II and BECN1 levels, and decreased p62 expression. These results demonstrated that one potential principal mechanism involved in the anti-apoptotic action of Rb1 might be increased autophagy in the aortic arteries of ApoE-/- mice.

In summary, the salient finding of this study is that ginsenoside Rb1 exerted an inhibitory effect on early atherosclerosis in ApoE-/- mice via decreasing body weight and food intake daily, upregulating the lipid levels of serum plasma, including those of TC, TG and LDL-C and HDL-C and reducing the atherosclerotic plaque area, suppressing inflammatory cytokines (levels of IL-1β, IL-6 and TNF-α) in the serum of ApoE-/- mice, changing the expression levels of BCL-2, BAX, cleaved caspase-3 and cleaved caspase-9 and weakening apoptosis associated with anti-inflammatory activity. Hence, all these effects against atherosclerosis were tightly associated with regulation of necrosis or apoptosis associated with anti-inflammatory activity. Additionally, the results found that ginsenoside Rb1 increased autophagy flux to inhibit apoptosis via cceleration of autophagy via promoting of transformation of LC3 from type I to type II in high-fat diet-induced atherosclerosis in ApoE-/- mice. This finding, along with those of the previous study, provides evidence that Rb1 promotes the process of autophagy to protect against atherosclerosis via regulating BCL-2 family-related apoptosis.

Therefore we speculate that a balance between apoptosis and autophagy occurs, which has a good inhibitory effect on coronary arteriosclerosis. The use of ginsenoside Rb1 to promote beneficial autophagy activation and to inhibit apoptosis may be a potential novel multi-targeted therapeutic strategy for atherosclerosis. However, the effects and mechanisms of ginsenoside Rb1 against atherosclerosis has not been clearly elaborated, and further researches will be carried on with, and in vitro tests has been going on to explore the potential mechanisms of Rb1 in inhibition imbalance of apoptosis and autophagy in atherosclerotic models in deed.
